# Upper-Limb Muscle Fatigability in Para-Athletes Quantified as the Rate of Force Development in Rapid Contractions of Submaximal Amplitude

**DOI:** 10.3390/jfmk9020108

**Published:** 2024-06-20

**Authors:** Gennaro Boccia, Paolo Riccardo Brustio, Luca Beratto, Ilaria Peluso, Roberto Ferrara, Diego Munzi, Elisabetta Toti, Anna Raguzzini, Tommaso Sciarra, Alberto Rainoldi

**Affiliations:** 1 Department of Clinical and Biological Sciences, University of Turin, 10043 Turin, Italy; gennaro.boccia@unito.it; 2 Neuromuscular Function Research Group, School of Exercise and Sport Science, University of Turin, 10126 Turin, Italy; luca.beratto@unito.it; 3 Department of Medical Sciences, University of Turin, 10126 Turin, Italy; alberto.rainoldi@unito.it; 4 Research Centre for Food and Nutrition (CREA-AN), 00178 Rome, Italy; ilaria.peluso@crea.gov.it (I.P.); elisabetta.toti@crea.gov.it (E.T.); anna.raguzzini@crea.gov.it (A.R.); 5 Rehabilitation Medicine Department, Italian Army Medical Hospital, 00143 Rome, Italy; robertoferrara85@gmail.com (R.F.); sciarratommaso@hotmail.com (T.S.); 6 Joint Veteran Defence Center, 00184 Rome, Italy; diego.munzi@gmail.com

**Keywords:** explosive strength, fatigability, sport, disability

## Abstract

This study aimed to compare neuromuscular fatigability of the elbow flexors and extensors between athletes with amputation (AMP) and athletes with spinal cord injury (SCI) for maximum voluntary force (MVF) and rate of force development (RFD). We recruited 20 para-athletes among those participating at two training camps (2022) for Italian Paralympic veterans. Ten athletes with SCI (two with tetraplegia and eight with paraplegia) were compared to 10 athletes with amputation (above the knee, N = 3; below the knee, N = 6; forearm, N = 1). We quantified MVF, RFD at 50, 100, and 150 ms, and maximal RFD (RFDpeak) of elbow flexors and extensors before and after an incremental arm cranking to voluntary fatigue. We also measured the RFD scaling factor (RFD-SF), which is the linear relationship between peak force and peak RFD quantified in a series of ballistic contractions of submaximal amplitude. SCI showed lower levels of MVF and RFD in both muscle groups (all *p* values ≤ 0.045). Despite this, the decrease in MVF (Cohen’s d = 0.425, *p* < 0.001) and RFDpeak (d = 0.424, *p* = 0.003) after the incremental test did not show any difference between pathological conditions. Overall, RFD at 50 ms showed the greatest decrease (d = 0.741, *p* < 0.001), RFD at 100 ms showed a small decrease (d = 0.382, *p* = 0.020), and RFD at 150 ms did not decrease (*p* = 0.272). The RFD-SF decreased more in SCI than AMP (*p* < 0.0001). Muscle fatigability impacted not only maximal force expressions but also the quickness of ballistic contractions of submaximal amplitude, particularly in SCI. This may affect various sports and daily living activities of wheelchair users. Early RFD (i.e., ≤50 ms) was notably affected by muscle fatigability.

## 1. Introduction

Manual wheelchair use involves many physical challenges for the upper limbs, mainly because of rapid force requirements and prolonged usage resulting in neuromuscular fatigue [1]. Additionally, the inefficiency of manual wheelchair propulsion as a mode of ambulation has been highlighted; indeed, in comparison to the legs, arm work is less efficient and more strenuous, resulting in a diminished physical capacity [2]. Neuromuscular fatigue due to extended wheelchair use may result in muscle coordination changes with a shift in joint power from the shoulder joint to the elbow [3]. This may result in excessive strain on elbow flexors and extensor muscles.

Neuromuscular fatigue is commonly evaluated as the exercise-induced decline in a muscle’s maximal force-generating capacity. The most widely used indicator is the isometric maximal voluntary contraction force (MVF) [4]. MVF is calculated over 3 to 5 s maximal isometric contractions. However, in sports and the daily living of para-athletes, such prolonged contractions are likely never adopted. This lack of task specificity could lead to an inaccurate estimation of the magnitude of neuromuscular fatigue.

In addition to MVF, the rate of force development (RFD) has recently gained popularity as a measure of explosive strength in various contexts. RFD is calculated from the ascending part of the force–time curve during an explosive contraction, either as a mean time-locked value or a maximal slope of force signal (RFDpeak). This measure has been studied extensively [5,6], and it is more functionally relevant than pure maximal strength [7,8]. RFD has been shown to be more sensitive than MVF in detecting chronic changes caused by factors such as disuse [9], strength training [10], and rehabilitation [11], as well as acute adjustments associated with exercise [12], muscle damage [13], and pain [14]. RFD, especially early RFD (≤50 ms), has been suggested to be largely influenced by neural mechanisms, mainly in relation to motor unit behaviour [15]. This physiological feature of RFD may explain why this variable is often more sensitive to changes than MVF [16], especially when the fatiguing task comprises rapid force production [17]. The analysis of muscle excitation in the first 50 ms of contraction employing high-density electromyography (HD-EMG) might provide even more insights into the causes of possible decrement of early RFD [17,18,19].

In this context, the protocol normally used to calculate RFD in submaximal amplitude contractions is the so-called RFD scaling factor (RFD-SF) [20,21,22,23]. The protocol consists of a series of fast, i.e., burst-like, contractions performed at different sub-maximal intensities (i.e., from 20 to 80% MVF) [24,25,26]. This means the participants aim to reach a submaximal force level as rapidly as possible. Such motor tasks mimic, in isometric conditions, the brief muscle excitation profiles typically observed in locomotion [27,28]. The adoption of RFD-SF has emerged as an informative measure to quantify the neuromuscular quickness of submaximal contractions [20,29,30,31]. For these reasons, we consider it more appropriate to detect muscle fatigability in real-life conditions [32]. 

This study aimed to compare neuromuscular fatigability of the elbow flexors and extensors between athletes with amputation (AMP, being above the knee, below the knee, or at the level of the forearm) and athletes with spinal cord injury (SCI, either paraplegia or tetraplegia) for maximum isometric muscle strength and RFD, and between time intervals for RFD and electromyographic signal amplitude. Comparing SCI and AMP para-athletes will help foster a more comprehensive understanding of disability sport performance, leading to better support, training, equipment, and inclusivity for athletes of all abilities.

## 2. Materials and Methods

### 2.1. Recruitment and Characteristics of Athletes

The study was conducted in accordance with the Declaration of Helsinki, and the protocol was approved by the Ethics Committee of the Italian Army Medical Hospital. All participants read and signed the informed consent form and knew they could withdraw at any time. A convenience sample of 20 athletes (10 with AMP and 10 with SCI) participating in two training camps (May and September 2022, Jesolo) for Italian Paralympic veterans was recruited for this study. The presence of SCI or AMP was applied as inclusion criteria. Data characterising the volunteers was collected through questionnaires [33]. The Joint Veteran Defence Center, Scientific Department, Army Medical Center, Rome, Italy, provided the health condition of each athlete. The main characteristics of athletes are depicted in Table 1. Wheelchair users typically adopted manual wheelchairs with propulsion assist devices in their leisure time.

### 2.2. Anthropometric Measurements

Body mass was measured on an electronic scale with an accuracy of 0.01 kg (Wunder RW 02, Trezzo sull’Adda, Italy) and calculated by subtracting the weight of the wheelchair/prosthesis and clothes (weighted separately) from total mass.

### 2.3. Neuromuscular Function Evaluation

#### 2.3.1. Setup

The participants utilised an identical setup to that previously employed by the authors [34]. In summary, the athletes with AMP were seated on a chair while athletes with SCI were seated on their wheelchair. All participants had their right arm flexed at a 90° angle from full extension and slightly abducted from the trunk (approximately 15°). The wrist was immobilised using non-elastic straps and a custom-built telescopic support. A strain gauge load cell (Model TF 022, CCt transducers, Turin, Italy) was connected to record compression and extension forces. The hand and forearm were positioned neutrally, and real-time visual feedback was displayed on a 48 cm × 27 cm computer screen. The force and EMG signals were sampled at a rate of 2048 Hz and converted to digital data using a 16-bit A/D converter (Sessantaquattro, OT Bioelettronica, Turin, Italy).

#### 2.3.2. High-Density Surface Electromyography

Two bidimensional HD-sEMG matrices of 32 electrodes each (4 rows × 8 columns, 8 mm inter-electrode distance, gold-coated; model: GR08MM0805, OT Bioelettronica, Turin, Italy) were placed over the right upper limb. The first was placed over the long head of the biceps brachii, and the second over the later head of the triceps brachii [35]. The reference electrode (24 mm, model: CDE-S. OT Bioelettronica, Turin, Italy) was placed on the acromion of the same limb; a strap ground electrode, dampened with water, was placed around the wrist. Before the array application, the skin was prepared to remove body hair, slightly abraded with an abrasive paste, and finally cleaned with water [36]. 

To ensure proper electrode–skin contact, the electrode cavities of the matrices were filled with 20–30 lL of conductive paste (Spes-Medica, Battipaglia, Italy). The electrode arrays were fixed with an extensible dressing. The EMG signals were amplified (gain 150), sampled at 2048 Hz, bandpass filtered (20–450 Hz, Butterworth 4th order) and converted to digital data with a 16-bit A/D converter (Sessantaquattro; OT Bioelettronica, Turin, Italy). Signals, in single-differential configuration, were visualised during acquisition and then stored on a personal computer using OT BioLab+ software version 1.5.5.0 (OT Bioelettronica, Turin, Italy) for further analysis. 

#### 2.3.3. Procedures

The experimenters paid particular attention to avoiding movement in the torso and shoulders during the execution of contractions. Additionally, participants were instructed to avoid activating their trapezius muscles during elbow flexion and leaning their body forward during elbow extension. All test sessions were conducted by the same investigators, and participants were given standardised verbal encouragement during the execution of maximal voluntary and rapid contractions.

The protocol started with a warm-up comprising 10 submaximal isometric contractions ranging from 20% to 80% of the perceived maximum force and the familiarisation with ballistic contractions (details to follow). Then, participants performed two maximal voluntary isometric contractions and the RFD-SF protocol before (PRE) and immediately after (POST) a graded arm cranking test, until task failure. The POST session started on average 3 ± 1 min after the end of the graded test.

To measure MVF at PRE, two 5 s maximal voluntary contractions were performed if the difference in MVF between the two trials was greater than 5%. 

**Figure 1:**
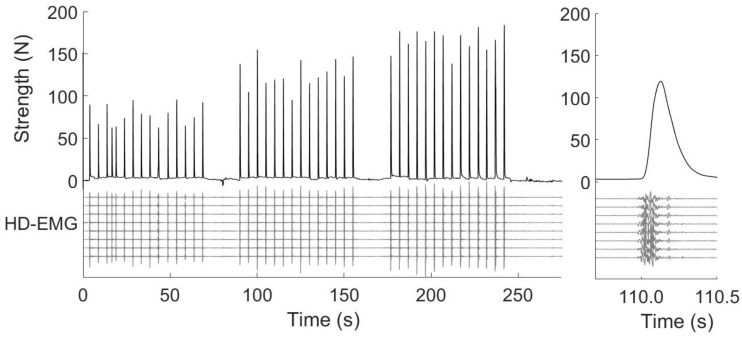
In the left panel**,** the upper part of the graph shows a representative example of the force signal recorded during the evaluation of the elbow extensor muscles of an amputee subject. The lower part of the graph shows the electromyographic (HD-EMG) signals from one of the four columns of electrodes that are part of the array placed on the triceps brachii (lateral head). In the right panel, the magnification of one repetition is plotted. As can be seen, the active phase of the muscle contraction lasts about 200 ms.

The RFD-SF protocol (Figure 1, left panel) began two minutes after the last maximal voluntary contractions. The original RFD-SF protocol requires the performance of 125 ballistic (burst-like, see Figure 1, right panel) isometric contractions across a full range of submaximal amplitudes [29]. The study utilised a shortened version of the original protocol, which consisted of at least 36 contractions and demonstrated reliable results [37]. Participants were instructed to perform 12 ballistic isometric contractions, interspersed by 5 s, at 20%, 40%, 60%, and 80% of their MVF, totalling 48 contractions. The levels of force required were randomised between participants and kept withing participants between PRE and POST. They were required to produce rapid contractions with peak forces reaching approximately ±10% of the target force. Each pulse was controlled by standardised acoustic cues. If a ballistic isometric contraction was not performed correctly, it was repeated. No changes in content were made. The computer screen displayed the range force as a horizontal band with a width of 20% MVF. Participants were instructed to perform each isometric torque pulse as quickly as possible and then relax immediately. The focus was on the speed of the contraction rather than the precision.

### 2.4. Incremental Arm-Cranking Graded Test

Athletes used an incremental graded arm-cranking ergometer to voluntary fatigue [23,24]. The purpose of the graded test was only to induce muscle fatigability; however, cardiorespiratory measurements were obtained using the wearable metabolic system K5 metabolimeter (COSMED, Rome, Italy) and under continuous heart rate monitoring (data not presented herein).

### 2.5. Signal Processing

#### 2.5.1. Onset Determination and Time Windows

The signal processing was performed using custom-written software in MATLAB (ver. 2023a, he MathWorks Inc., Natick, MA, USA). Force onsets were automatically detected through a hand-customised MATLAB code [7,38]. In the case that contractions presented countermovement or pretension, they were removed from the analysis. On average, no more than 3 contractions were removed from voluntary contraction at each time point (i.e., PRE and POST) for each subject. The EMG time windows were calculated from EMG onset, whilst force time windows were calculated from force onset. Therefore, EMG and force time windows (i.e., the windows of 50, 100, and 150 ms) were shifted by the time difference between EMG and force onsets (i.e., electromechanical delay), which we arbitrarily set to 15 ms based on previous studies on voluntary contractions [17].

#### 2.5.2. Force Signal

The force signals were low-pass filtered at 100 Hz using a fourth-order zero-lag Butterworth. MVF was measured from the 5 s maximum voluntary contractions, and it was defined as the highest force over the two trials performed at PRE and the single trial performed at POST. All RFD parameters were calculated from the burst-like contractions of the RFD-SF protocol. RFD (Δforce/Δtime) was estimated at 50, 100, and 150 ms (defined as RFD50, RFD100, RFD150). Maximum RFD (RFDpeak) was calculated as the maximum first derivative of the force signal from the onset of contraction using a 20 ms moving average window [39].

To calculate the RFD-SF, the force signal was pre-processed using an overlapping moving window of 0.1 s [20,32,40]. The use of a moving window was preferred over a 5 Hz low-pass filter to avoid introducing aberrations in the signals, which are typically evident as a force signal below zero just before the onset of contraction. Next, the RFD signal was obtained by computing the first derivative of the force signal. The peak force and RFDpeak (the local maximum of the RFD signal) were determined for each ballistic contraction. The RFD-SF was calculated by determining the linear regression slope between peak force and peak RFD for each contraction. RFD-SF measures how RFD scales with force in a range of submaximal contractions, providing a quantification of quickness across a span of intensities. Outliers were identified and removed using the Cook distance methodology to improve the fit of the linear regression [41].

#### 2.5.3. High-Density Surface Electromyography

EMG channels with excessive noise or artefacts were removed after visual analysis. Then, we identified the innervation zone for each matrix of electrodes and selected the channels with propagating action potentials. Single-differential EMG signals were calculated for each column and visually inspected. Four to eight single-differential EMG channels with clear motor unit action potential propagation without shape change from the nearest innervation zone to the distal tendon were chosen for the analysis.

The amplitude of voluntary HD-sEMG signals was assessed as the root mean square (RMS) across all available channels. RMS calculated at 50, 100, and 150 ms from EMG onset (defined as RMS50, RMS100, RMS150) was then averaged across channels to obtain a single value for each muscle. This procedure produces more reliable results in voluntary and evoked contractions [42].

### 2.6. Statistical Analysis

Statistical analysis was performed in R (ver. 3.5.2, R Development Core Team, 2009) and JASP (JASP team, version 0.18.3). First, we adopted a series of repeated-measure ANOVAs to compare the trend of each mechanical and EMG variable in time (PRE vs. POST), between conditions (SCI vs. AMP), and between time intervals (50, 100, and 150 ms). 

Then, to answer the main experimental question, we analysed the peakRFD with multilevel mixed linear regression analysis through the package lme4 Version 1.1.19. Linear mixed-effects models are particularly suitable in this experimental design, as participants performed dozens of contractions with each muscle group, and the model accounts for such a hierarchical data structure. We adopted the peak force reached in each contraction, time (PRE vs. POST), muscle group (elbow flexors vs. extensors), and condition (SCI vs. AMP) as fixed factors, and we considered the random intercept over participants and the random slope of muscle group (as each muscle group, within each participant, may have different levels of strength):

peakRFD ~ peak force × time × muscle × condition + (muscle|subjects)

Paired, two-tailed Student’s *t*-tests were used to compare the other parameters between PRE vs. POST. The Kolmogorov–Smirnov normality test was used to assess distribution normality. Post hoc analysis was adjusted with Bonferroni corrections. The level of statistical significance was set to *p* < 0.05. In graphs, data are reported as mean and 95% confidence intervals (C.I.). The effect size in ANOVA analysis was reported as partial eta squared η^2^. The magnitude of the difference between PRE vs. POST was calculated as Cohen’s d effect size. Threshold values for effect size statistics were <0.2, trivial; ≥0.2, small; ≥0.5, moderate; ≥0.8, large; and ≥1.4, very large.

## 3. Results

### 3.1. Incremental Arm-Cranking Graded Test

AMP reached greater peak power output than SCI (AMP: 128.0 ± 6.1 W, SCI 88.0 ± 9.0 W, *p* < 0.001). AMP also reached greater peak HR (AMP: 171.5 ± 5.4 beats/min, SCI 131.7 ± 14.5 beats/min, *p* < 0.05) and VO_2peak_ (AMP: 34.4 ± 2.8 mL/kg/min, SCI 23.1 ± 1.2 mL/kg/min, *p* < 0.001).

### 3.2. Neuromuscular Function Differences between AMP and SCI

**Figure 2:**
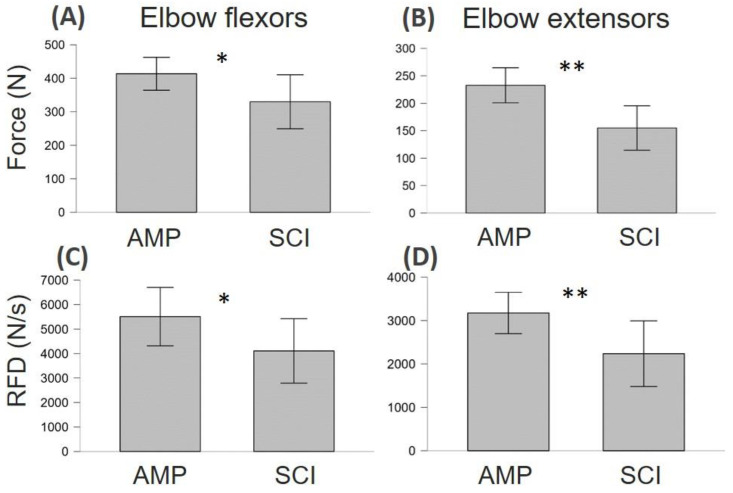
The level of maximum voluntary force (MVF) and rate of force development (RFD) of the elbow flexor and extensor muscles in athletes with amputation (AMP) and athletes with a spinal cord injury (SCI) for elbow flexors (**A**,**C**) and elbow extensors (**B**,**D**). * *p* < 0.05, ** *p* < 0.01.

As shown in Figure 2, SCI showed lower levels of maximum strength and explosive force capacity compared to AMP. In particular, SCI showed lower MVF in both elbow flexor muscles (d = 0.460, *p* = 0.045) and extensor muscles (d = 0.700, *p* = 0.003). Similarly, RFD in SCI was lower in both elbow flexor muscles (d = 0.400, *p* = 0.045) and extensor muscles (d = 0.500, *p* = 0.032).

### 3.3. Fatigability Effect on Neuromuscular Function

**Figure 3:**
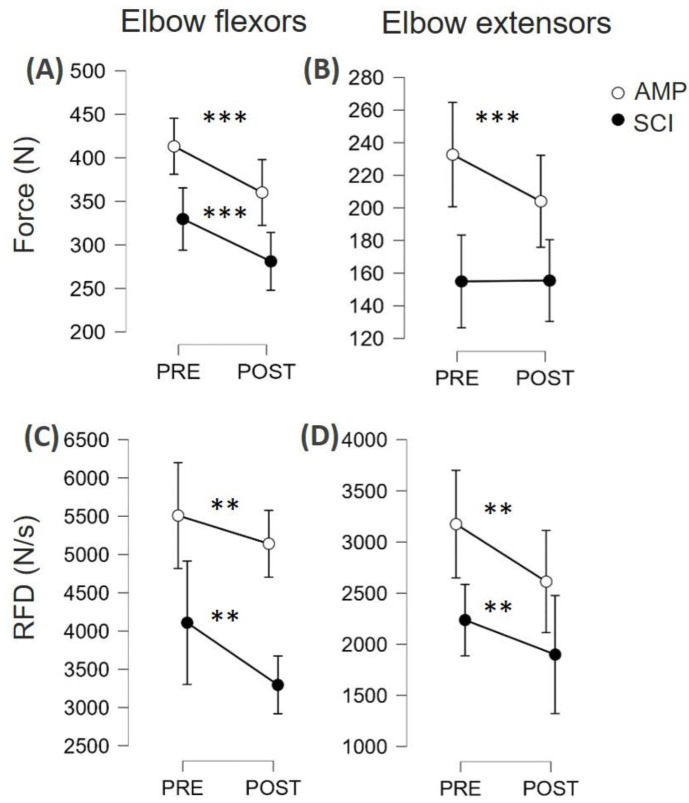
Maximum voluntary force (MVF) and rate of force development (RFDpeak) of the elbow flexor and extensor muscles in athletes with amputation (AMP) and athletes with a spinal cord injury (SCI) are reported for PRE and POST for elbow flexors (**A**,**C**) and elbow extensors (**B**,**D**). ** *p* < 0.01, *** *p* < 0.001.

**Figure 4:**
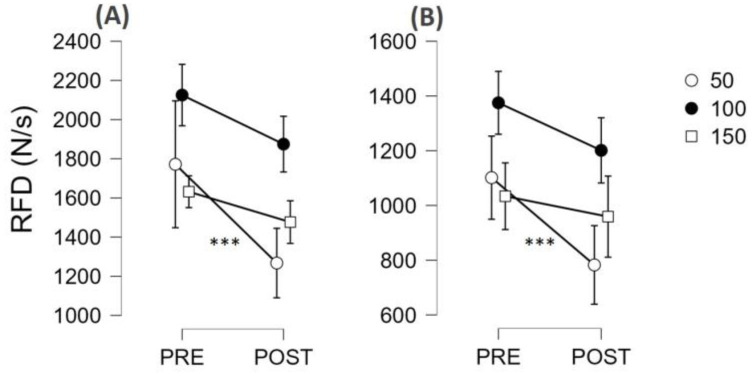
Rate of force development (RFD) values (mean and 95% CI) before (PRE) and after (POST) a maximal arm ergometer test (fatiguing task) are reported. Values are shown for the elbow flexor (**A**) and elbow extensor (**B**) muscles and are reported separately for 50, 100, and 150 ms time intervals. As the condition did not emerge as a significant factor, the two groups (AMP and SCI) were merged. *** *p* < 0.001.

As can be seen in Figure 3A,B, there was a significant effect of the fatiguing task on MVF (F = 19.4, η_p_^2^ = 0.520, *p* < 0.001) both in SCI and AMP. There was no statistically significant interaction between the groups in fatigue susceptibility (group × time interaction: F = 1.3, η_p_^2^ = 0.067, *p* = 0.269). However, as can be seen in Figure 2D, SCI appeared to have no signs of fatigue on the elbow extensor muscles, as force levels remained constant (PRE 155 ± 56 N; POST: 155 ± 69 N). As can be seen in Figure 4C,D, there was a moderate effect of the fatiguing task on RFD (F = 12.0, η_p_^2^ = 0.401, *p* = 0.003). In fact, RFD force decreased in both groups (*p* < 0.01 for all muscle groups, see Figure 4C,D). There was no statistically significant interaction between groups in fatigue susceptibility (group × time interaction: F = 1.3, η_p_^2^ = 0.007, *p* = 0.719). 

The time-locked analysis of RFD showed that there was no interaction with the condition (SCI vs. AMP, all *p* values greater than 0.185); therefore, the results are presented by merging the two groups together (Figure 4). There was not an interval × muscle × time interaction (*p* = 0.576), but there was an interval × time interaction (F = 11.2, η_p_^2^ = 0.385, *p* < 0.001) suggesting that the two muscle groups behaved similarly, but some time intervals were more susceptible to fatigue than others. Post hoc analysis showed that RFD50 showed the greatest decrease (d = 0.741, *p* < 0.001), RFD100 showed a small decrease (d = 0.382, *p* = 0.020), and RFD150 did not decrease (*p* = 0.272). The post hoc results within each muscle group are reported in Figure 4.

**Figure 5:**
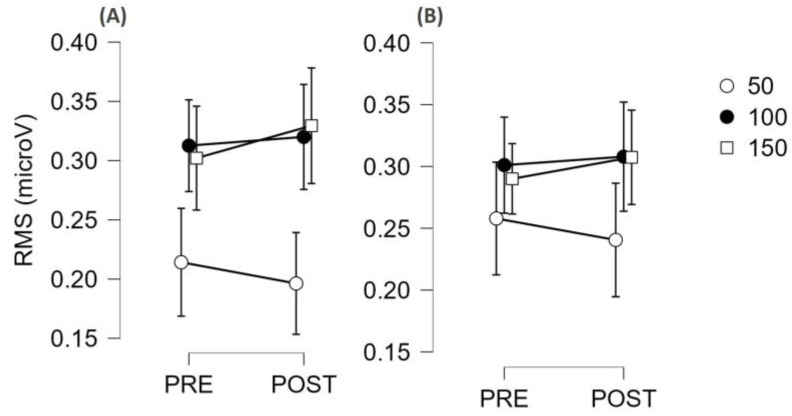
Electromyographic signal amplitude (RMS) values (mean and 95% CI) before (PRE) and after (POST) a maximal arm ergometer test (fatiguing task) are reported. Values are shown for the elbow flexor (**A**) and elbow extensor (**B**) muscles and are reported separately for 50, 100, and 150 ms time intervals. As the condition did not emerge as a significant factor, the two groups (AMP and SCI) were merged.

The time-locked analysis of RMS showed that there was not any interaction with the condition (SCI vs. AMP, all *p* values greater than 0.122); therefore, the results are presented by merging the two groups together (Figure 5). There was no interval × muscle × time interaction (*p* = 0.922), but there was an interval × time interaction (F = 7.0, η_p_^2^ = 0.293, *p* = 0.003), suggesting that the two muscle groups behaved similarly, but some time intervals were more susceptible to fatigue than others. However, post hoc analysis did not detect any significant differences, even though the RMS50 tended to decrease with time and RMS100 and RMS150 tended to increase with time (see Figure 5).

**Figure 6:**
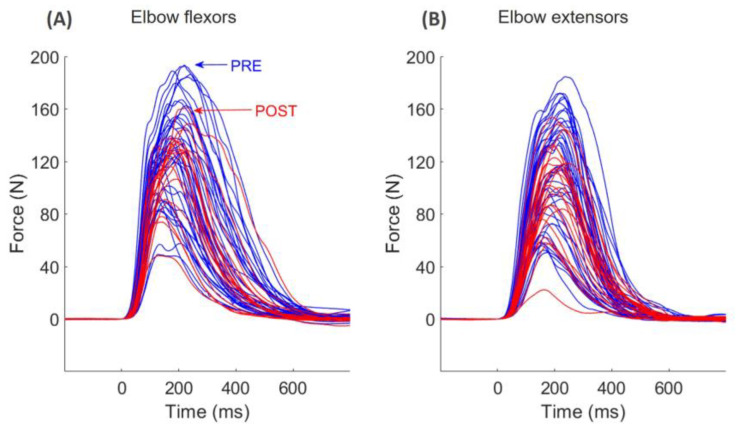
Representative example of the force recorded during all explosive muscle contractions performed before (blue) and after (red) the fatiguing task in an amputee subject in elbow flexors (**A**) and extensors (**B**).

At POST, participants reached lower levels of force (−12%, d = 0.257, *p* = 0.002) during the rapid burst-like contractions compared to PRE (see Figure 6, F = 12.6, η_p_^2^ = 0.414). There was no interaction with the muscle group or condition (*p* = 0.837). 

**Figure 7:**
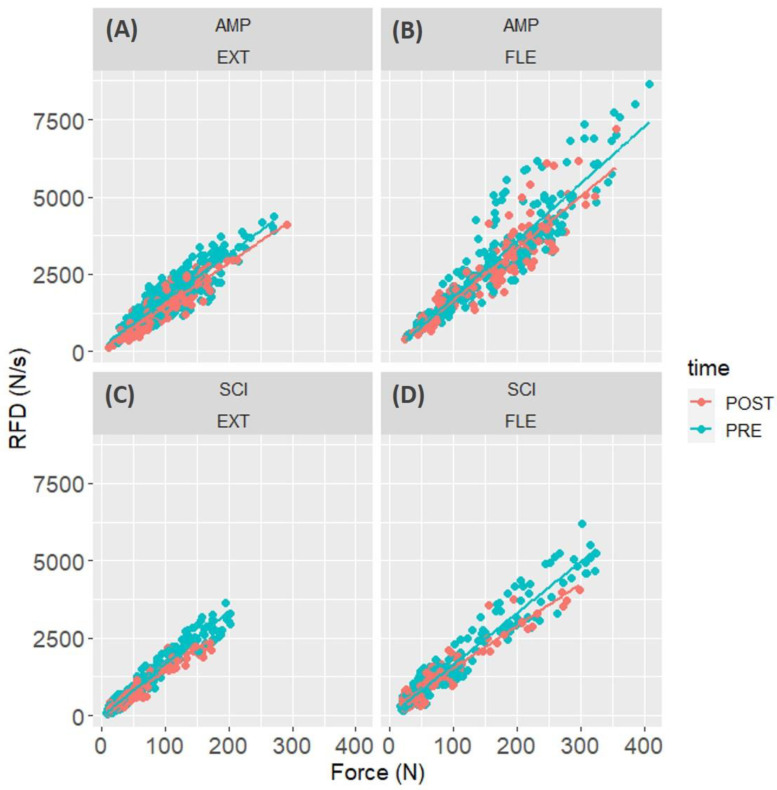
Peak rate of force development (RFD) is plotted against the peak force reached in each contraction of the RFD-SF protocol before (PRE) and after (POST) the fatiguing task. Values are shown for the elbow extensor (EXT, (**A**,**C**)) and elbow flexor muscles (FLE, (**B**,**D**)) separately for subjects with amputation (AMP, (**A**,**B**)) and with spinal cord injury (SCI, (**C**,**D**)).

The linear hierarchical model analysing RFD in the RFD-SF protocol showed that there was a peak force × time interaction (F = 72.3, *p* < 0.0001), meaning that the linear relationship between the peak force and RFD reached in each contraction changed between PRE and POST (Figure 7). Indeed, the estimate of RFD-SF merging all participants, i.e., the slope of the linear regression between peak force and peak RFD of all contractions and participants, decreased after the fatiguing task (Figure 7). There was also a significant peak force × time × condition (F = 20.8, *p* < 0.0001), showing that SCI had a larger decrease in RFD-SF than AMP. In particular, RFD-SF decreased from 15.0 to 14.1 in elbow extensors of AMP (Figure 7A), from 18.4 to 16.8 in elbow flexors of AMP (Figure 7B), from 17.0 to 14.3 in elbow extensors of SCI (Figure 7C), and from 16.6 to 13.6 in elbow flexors of SCI (Figure 7D).

## 4. Discussion

This study aimed to assess neuromuscular fatigue by comparing rapid force production between athletes with amputation (AMP) and spinal cord injury (SCI) in their elbow flexors and extensors. The present experimental setup involved a series of rapid isometric contractions at various submaximal intensities, mimicking brief muscle excitation profiles observed in various daily life activities. This approach aimed to provide a more functionally relevant measure of muscle fatigue. The participants, comprising AMP and SCI athletes, underwent neuromuscular function evaluations before and after a graded arm cranking test to voluntary fatigue. We found that (1) at PRE, SCI had lower MVF and RFDpeak values than AMP (all d values were ≈ 0.4–0.5); (2) after the fatiguing task, SCI and AMP showed a similar decrease of both MVF and RFDpeak (all d values were ≈0.4); (3) in the time-locked analysis of RFD, both groups showed a larger reduction in RFD50 (d = 0.7) compared to RFD100 (d = 0.4) and RFD 150 (not affected); (4) overall, the amplitude of HD-EMG did not change in all time intervals (Figure 5); (5) at POST, the RFD-SF decreased, more in SCI than in AMP, meaning that the quickness of ballistic contractions of submaximal amplitude decreased after the fatiguing task (Figure 6 and Figure 7).

### 4.1. Differences and Similarities between SCI and AMP

The SCI athletes exhibited lower MVF and RFD levels than AMP athletes in both elbow flexors and extensors (Figure 2). These findings underscore the differential impact of spinal cord injury versus limb amputation on the neuromuscular function of the upper limb. Additionally, both groups demonstrated decreased RFD and MVF following the fatiguing task (Figure 3), suggesting similar susceptibility to fatigue across populations, albeit by varying degrees. Interestingly, while both groups exhibited fatigue, SCI athletes showed no signs of fatigue in the elbow extensor muscles, as force levels remained constant. This observation could suggest differential fatigue patterns between muscle groups or adaptations specific to the SCI population. More likely, many SCI athletes in our sample could barely activate their elbow extensor (as can be seen from the force produced, see Figure 2). Therefore, that muscle group probably did not perform sufficient muscle work during the fatigue test to generate signs of muscle fatigue. In other words, elbow extensors did not fatigue through exercise because they were not activated enough to disturb the metabolic condition. Further investigations are warranted to elucidate these findings.

### 4.2. Fatigability Expressed as Reduction of MVF or RFDpeak

The study highlighted the importance of task specificity in evaluating neuromuscular fatigue, particularly in para-athletes whose daily activities may not involve prolonged maximal contractions. While MVF is a standard measure, it may not accurately reflect the fatigue experienced during more functional tasks. RFD, especially early RFD (≤50 ms from the contraction onset), has been suggested to be more sensitive to changes in neuromuscular function due to fatigue [16,43,44], potentially because of its reliance on neural mechanisms related to motor unit behaviour [45]. The time-locked analysis of RFD revealed a significant decrease across time intervals, with the greatest decrease observed at RFD50, followed by a smaller decrease at RFD100, and no change at RFD150. This pattern suggests that the early phase of force production is particularly susceptible to fatigue, which aligns with previous research highlighting the importance of early rapid force generation in the fatigued condition [17]. Of note, those reductions were similar in SCI and AMP, suggesting the two groups of para-athletes had similar susceptibility to fatigue induced by an incremental test at the arm ergometer.

### 4.3. Contraction Quickness in Ballistic Contractions of Submaximal Amplitude

The adoption of RFD-SF to quantify neuromuscular quickness [46] has been applied to detect asymmetries [34,47,48], ageing [29,49], and neuromuscular disorders [50]. Nevertheless, using the RFD-SF, two previous studies failed to detect neuromuscular [30] or mental fatigue [49]. In the present study, we demonstrated for the first time that RFD-SF is susceptible to neuromuscular fatigability induced by an incremental arm-cranking test. Beyond the fact that the previous study was conducted on the lower limbs [30], the shorter recovery in the present study (≈3 min) compared to the previous one (5–8 min) may have limited the recovery of fatigue, thus allowing its detection through RFD-SF protocol. 

Here, we found that the capacity to perform ballistic contractions of submaximal amplitude is altered in the presence of fatigue (Figure 7). While the present study cannot identify the physiological cause for this impairment (as the HD-EMG amplitude did not change over time, Figure 5), it is possible to speculate on the consequences of this finding. Neuromuscular fatigability has always been detected using maximal contractions because it was operationally defined as a decrease in muscle strength/power [4]. However, the fact that the maximal strength decreases does not necessarily mean that the strength available in rapid contractions of submaximal force levels decreases. Therefore, it would be relevant to directly measure the reduction in capacities of performing short rapid contractions, much shorter than those required to measure MVF, like the ones typically used in the stroke to push a wheelchair. With the RFD-SF protocol, we demonstrated a quickness reduction in muscle contraction modality that is more relevant for both athletic performance and daily activities, particularly among para-athletes. Furthermore, we demonstrated that people with spinal cord injury might be more susceptible to fatigability in this specific task (Figure 7). The present findings contribute to our understanding of neuromuscular fatigue in para-athletes and underscore the importance of task-specific assessments. Future research should explore the physiological roots of this behaviour, considering the unique challenges posed by different impairments.

### 4.4. Electromyographic Parameters

The muscle activation, measured as the RMS of the HD-EMG signal, was calculated in 50 ms time intervals from the onset of muscle contractions. The fact that the amplitude of the EMG signal did not decrease (Figure 5) can be explained in two ways: (1) there was no decrease in the neural command under fatigue conditions; (2) there was a decrease in the neural command, but the amplitude estimation was affected by confounding factors intrinsic to the electromyographic evaluation (such as the phenomenon of amplitude cancellation) that overestimate the amplitude of the signal under fatigue conditions. In any case, the fact that the amplitude of the EMG signal did not decrease suggests that there was no appreciable decrease in neural activation during the explosive contractions and, therefore, central fatigue was limited. Consequently, the decrease in rapid force production that herein reported is probably due to factors of peripheral origin (e.g., decreased muscle contractility). 

### 4.5. Limitations

The sample size of the present study is small in absolute terms. However, considering the small number of people in the reference population, i.e., Italian paralympic veterans, which comprises a few dozen people, the number of participants recruited in the present study was considerable. Furthermore, the presence of two wheelchair users in the group with amputation might have slightly confused the results. It would have been useful to test the fatigability of the shoulder muscles as well. However, we had to reduce the number of muscles tested in order to reduce the testing time, especially at POST. This is because increasing the number of muscles tested would have increased the recovery time, i.e., the time between the end of the exercise and the test, thus invalidating the measures of fatigue. Furthermore, measuring the actual torque instead of force would have improved the reliability of our results.

## 5. Conclusions

We firstly demonstrated that muscle fatigability impacts not only maximal force expression, i.e., maximal strength (i.e., MVF) and maximal quickness (RFDpeak), but also the quickness of ballistic contractions of submaximal amplitudes, especially in para-athletes with spinal cord injury. Consequently, the effects of muscle fatigability can be seen also during many sports and daily living activities of wheelchair users. We also found that early RFD, i.e., the quickness of the first 50 ms of muscle contraction, were particularly affected by muscle fatigability, further highlighting the importance of evaluating the RFD with the adoption of time-locked intervals instead of only RFDpeak. As expected, lower muscle strength and explosive capacity were observed in para-athletes with spinal cord injury compared to those with lower limb amputation; however, the two groups of para-athletes showed an equal decrease in strength despite starting from lower initial strength levels.

## Figures and Tables

**Table 1:** Characteristics of athletes.

	AMP	SCI
Age, years	43.3 ± 2.8	44.6 ± 3.4
Lesion level (incomplete for SCI, prosthesis for AMP)	Above knee, n = 3 Below knee, n = 6 Forearm, n = 1 Wheelchair users (2/10)	Tetraplegia (C6-C7), n = 3 Paraplegia (T7-T12), n = 7 Wheelchair users (10/10)
Sport practiced	Athletics, Basketball, Cycling, Sitting volleyball, Swimming, Tennis	Athletics, Archery, Hand-bike, Sitting Volleyball, Swimming, Power Soccer
Training/h week	5.3 ± 0.9	5.2 ± 1.0
Television/h week	12.0 ± 3.6	11.8 ± 2.6
Screening/h week	27.9 ± 7.7	22.4 ± 4.1
Sleeping/h day	7.1 ± 0.3	7.6 ± 0.2
Neurogenic bowel, %	-	40%
Neurogenic bladder, %	-	40%

AMP: athletes with amputation, SCI: athletes with a spinal cord injury.

## Data Availability

Data are unavailable due to privacy and ethical restrictions.
